# Bilateral Facial Palsy as the Onset of Neurosarcoidosis: A Case Report and a Revision of Literature

**DOI:** 10.3390/neurosci3020023

**Published:** 2022-05-29

**Authors:** Chiara Gallo, Letizia Mazzini, Claudia Varrasi, Domizia Vecchio, Eleonora Virgilio, Roberto Cantello

**Affiliations:** 1 Department of Neurology, University of Eastern Piedmont, Maggiore della Carità Hospital, 28100 Novara, Italy; 20032512@studenti.uniupo.it (C.G.); mazzini.letizia5@gmail.com (L.M.); claudia.varrasi@libero.it (C.V.); domizia.vecchio@gmail.com (D.V.); roberto.cantello@med.uniupo.it (R.C.); 2 Ph.D. Program in Medical Sciences and Biotechnologies, Department of Translational Medicine, University of Eastern Piedmont, 28100 Novara, Italy

**Keywords:** neurosarcoidosis, sarcoidosis, cranial neuropathy, facial diplegia, lymph node biopsy

## Abstract

Unilateral facial nerve palsy (FNP) is one of the most common cranial mononeuropathies. Among rare etiologies, neurosarcoidosis (NS) can cause bilateral involvement (both recurring and simultaneous) only in 15% to 25% of cases. The rarity of this systemic disease and its clinical heterogeneity, due to granulomatous inflammation that may affect many anatomic substrates, frequently make the diagnosis a real challenge for the clinician. Based on laboratory and instrumental tests, a careful diagnostic algorithm must be adopted to avoid misdiagnosis and delay in treatment. We present a 52-year-old woman with an acute onset of unilateral right FNP, rapidly developing contralateral involvement (simultaneous bilateral FNP). Lung findings pointed towards a systemic disease, and then lymph node biopsy confirmed NS. Corticosteroid therapy was started. After three years of follow-up, the patient is still in remission with a low prednisone dose. We discuss the differential diagnosis of bilateral FNP, focusing on clinical presentation, diagnosis, and treatment of NS. We have performed a literature revision, confirming bilateral FNP, outside Heerfordt syndrome, to be rare and sometimes represent the only neurological manifestation of NS onset.

## 1. Introduction

Facial nerve palsy (FNP) is a frequent cranial mononeuropathy [1]. Overall, 70% of cases are idiopathic, not recognizing a specific etiology, best known as Bell’s palsy. This condition may leave a residual facial weakness, but it is not a life-threatening condition. Furthermore, many different traumatic, iatrogenic, infectious, and systemic diseases can manifest as FNP more rarely than Bell’s palsy [1,2,3]. Additionally, bilateral FNP is even more uncommon, ranging from 0.3% to 2% of all FNP cases, and recognizes fewer identifiable causes [4], which could be amenable to prompt medical management or surgical approach. The ruling out of all life-threatening diseases, such as leukemia or Guillain-Barre syndrome (GBS), is mandatory and prioritizes workups [3,4,5]. The diagnostic algorithm includes targeted laboratory and instrumental findings (such as brain MRI), depending upon then history, which may lead to identifying even the rarest causes of simultaneous presentation throughout a real diagnostic challenge. Cranial neuropathy is the most common manifestation of neurosarcoidosis (NS), with cranial nerves II, VII, and VIII being the most frequently affected [6] and a high frequency of subclinical leptomeningeal involvement. About one-third of FNP are bilateral and could be recurrent (RBFP) or simultaneous (SBFP). SBFP is defined as the involvement of the opposite side within 30 days of the onset of the first side [6,7]. Facial diplegia in NS is also described as SBFP/RBFP, associated with fever and the involvement of parotid glands and uveitis (also known as “Heerfordt’s syndrome”) [8,9,10,11,12]. Since NS is a rare disease, the recognition of bilateral FNP may represent a red flag for the clinician to direct early diagnosis and intervention. Thus, we present the case of a 52-year-old Caucasian woman with SBFP and a review of the existing literature regarding bilateral facial palsy/plegia as a manifestation of NS.

## 2. Case Report

**Figure 1:**
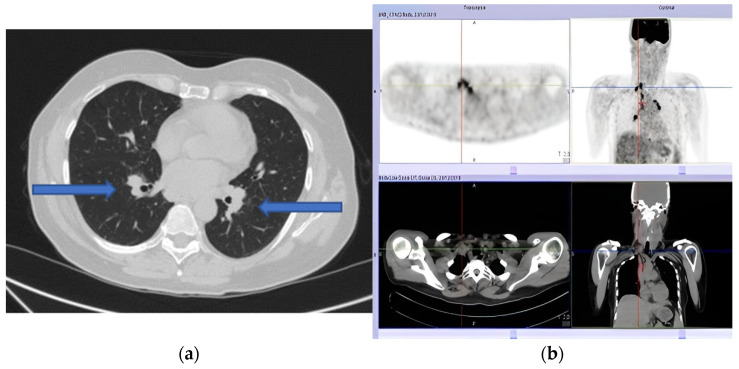
(**a**) Pulmonary computed tomography (CT) scan showing multiple bilateral lymphadenopathies. (**b**) Positron emission tomography (PET) showing multiple adenopathies with a high metabolic component.

Our patient presented to the Emergency Department with House–Brackmann scale grade V (corresponding to severe dysfunction, barely perceptible motion, and asymmetry at rest) right peripheral FNP. Her past medical history included situational depression with anxiety and mild tension-type headache occurring since adolescence. She was on no medications, and she denied any tobacco, alcohol, or drug abuse, exposition to toxic agents, or recent infection. Vital parameters were normal. Lungs auscultation and abdomen evaluation were unremarkable. Herpes Zoster infection was first excluded by searching for vesicles or scabbing. Then, a complete neurological examination showed right tongue deviation, impaired sensation to the right side of the face, and an alteration to taste sensation. A brain CT scan ruled out a cerebrovascular accident of posterior circulation. Subsequent brain MRI with contrast was normal. A complete blood count and a comprehensive metabolic panel showed no abnormality. A routine chest X-ray showed multiple bilateral lymphadenopathies confirmed by chest CT (Figure 1a). We excluded occult neoplasia, performed a mammography and breast ultrasound (BI-RADS 1 bilaterally), and planned a total body PET scan (Figure 1b).

**Figure 2:**
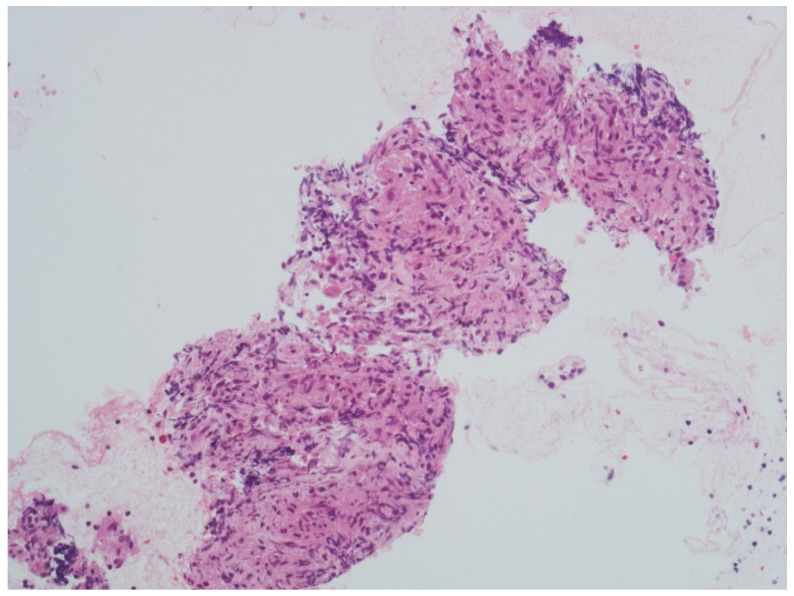
Parahilar lymph node biopsy showing epithelioid macrophages in granulomatous aggregation (courtesy of Professor R. Boldorini Division of Pathology, Department of Health Science, University of Piemonte Orientale (UPO), Novara, Italy)**.** Scale bar: 100 μm (40× magnification).

After a week, she presented with left peripheral FNP, bilateral hearing loss, limitation in left gaze, expression of left sixth cranial nerve palsy, and liquid dysphagia. The examination confirmed the presence of cranial multineuritis. Following readmission, a lumbar puncture was performed with cerebrospinal fluid (CSF) analysis showing a slight increase in cell count (17 cells/mm^3^—monocytes), normal protein (31 mg/dL), and glucose level (70 mg/dL); malignant cells were not seen on cytology. The PET scan showed multiple supraclavicular, paratracheal, epiaortic, paraesophageal, and parahilar adenopathies with a high metabolic component. Serological tests, including ANCA, ANA, HIV antibody test, tuberculosis, and B.Borrelia serology, were negative, but the sedimentation rate of the erythrocytes (ESR) was 24 mm/h. Flow cytometry was not performed. Based on clinical presentation and exam results, the suspect of a granulomatous disorder was made, and corticosteroid therapy (1 mg/kg of prednisone–55 mg/day) was started with a partial clinical benefit over four days. A needle biopsy was performed via bronchoscopy and showed epithelioid macrophages in granulomatous aggregation to characterize the lymph node alterations (Figure 2).

The histological finding was compatible with sarcoidosis. The serum ACE value was increased (67.7 U/L—normal range 8.0–52.0), and the CSF-ACE level was not performed. The clinical presentation, the evidence of central nervous system (CNS) inflammation (CSF findings), typical histological findings of granulomatous inflammation of tissue biopsy in at least one extraneural organ, and the exclusion of other causes confirmed a diagnosis of probable neurosarcoidosis in pulmonary sarcoidosis grade I. She improved on this regimen with partial resolution of her facial palsies after six weeks of steroid therapy (right paralysis: House–Brackmann grade III; left paralysis: grade II) and complete hearing loss regression. Her steroids were slowly tapered after eight weeks of therapy, and she continues to do well with 12.5 mg of prednisone after three years of follow-up. After a pneumological examination, immunosuppressant treatment with methotrexate was recommended to reduce and discontinue steroid therapy.

## 3. Literature Review

### Search Strategy and Selection Process

According to the guidance on narrative reviews, we conducted a literature search in PubMed, up to 17 March 2022, combining the main terms “facial diplegia neurosarcoidosis”, ((“bi-lateral facial paralysis”) OR (“bilateral facial palsy”) OR (“bilateral seventh nerve palsy”)) AND ((“neurosarcoidosis”) OR (“sarcoidosis”)). Clinical studies, case reports, and case series reporting NS patients with acute bilateral recurrent or simultaneous FNP published in English, Dutch, Spanish, and French between 1970 and March 2022 were considered for inclusion. Two authors (CG and EV) independently screened seventy titles, selecting sixty abstracts and retaining fifty-one full texts of all relevant articles. Three articles were excluded, published between 1970 and 1980, due to difficulty finding the full-text and only partial clinical information. Finally, twenty-one articles were selected [8,13,14,15,16,17,18,19,20,21,22,23,24,25,26,27,28,29,30]. Two articles conducted in the same center had only partial information regarding patients with BFP and, therefore, could not be included in the final results [31,32]. Data on study characteristics, demographic features, clinical manifestations, treatment, and outcome are reported below in Table 1, Table 2 and in Table S1 (reported in Supplementary Materials).

When analyzing the literature, the diagnosis of pulmonary sarcoidosis was defined according to the known radiological criteria by Siltzbach (from “Grade 0—no radiological findings” to “Grade 4: pulmonary fibrosis”) [33]. Diagnostic criteria for NS (as by Zajicek et al.; Marangoni et al.), used by various authors before 2018, have been revised. NS cases were re-classified as possible, probable, and definite according to revised The Neurosarcoidosis Consortium Consensus Group (NCCG) diagnostic criteria (2018) [34]. The pathologic confirmation of systemic granulomatous disease with biopsy, consistent with sarcoidosis, was also analyzed. NS diagnosis, based on the Forearm Kveim test biopsy [14], was redefined as “possible”: this test is no longer used in clinical practice and is not explicitly included in the latest diagnostic update criteria [34]. In one case, biopsy resulted negative, and we redefined the patients as “possible NS” [15]. In this patient, to note, the tissue sampling was performed one month after the steroid therapy, possibly affecting the result. In addition, two cases of NS were excluded [35,36], respectively, for lacking diagnostic data on NS according to NCGG diagnostic criteria [34]. Therapy was classified as first-line, second-line, or third-line therapy. First-line therapy corresponds to corticosteroid treatment, second-line treatment consists of immunosuppressive therapy with methotrexate, azathioprine, mycophenolate mofetil, and hydroxychloroquine, and third-line therapy either consists of tumor necrosis factor-alpha inhibitors (principally infliximab) and B-cell targeted therapy [37], according to the last ESR clinical practice guidelines [38]. The prognosis was established using the House–Brackmann Scale (H-B Scale), and remission was defined as a complete improvement without residual symptoms.

## 4. Results

Overall, excluding our patient, 17 adults with bilateral FNP as the onset of NS (not showing Heerfordt’s syndrome signs) have been reported in the literature from January 1970 to March 2022. Table 1 and Table 2 shows the demographic and clinical characteristics of these patients. Kidd reported 12 bilateral and simultaneous FNP patients in a population of 166 (7%) “highly probable” NS patients, according to the WASOG sarcoidosis organ assessment instrument criteria. No other data for specifically these 12 patients were available in the papers; therefore, they were not considered in Table 1 and Table 2 [31,32]. Among the NS patients, the majority were females (eight; 73%), and three were males (gender was not reported in six cases). The median age at presentation was 39 years (ranging from 24 to 62 years old). Fourteen patients (82%) presented with SBFP involvement: simultaneous diplegia (SBFD) was detected in five cases. RBFP were only observed in three patients. According to the NCCG diagnostic criteria, the diagnosis was “probable NS” in six cases. Only in one patient in which a sural nerve biopsy was performed was it possible to define a diagnosis of “definite NS” [26]. Three “possible” diagnoses were found. In seven patients only described as “NS,” the revision of diagnosis was impossible due to lacking information. We will proceed by showing the characteristics of “definite,” “probable,” and “possible” NS. A patient presented with a “probable isolated NS”; systemic sarcoidosis was detected in nine patients, all with pulmonary sarcoidosis (grade I was observed in 80% of the cases; grade II in 20%). Multiorgan involvement was found in nine patients (53%) with systemic (weight loss and soft tissue lymphadenopathy—three), renal (two), ocular (two), upper respiratory tract (URT) (two), skin (two), and hematological manifestations (one). In all cases, bilateral facial nerve involvement did not appear in an already diagnosed sarcoidosis and was one of the first clinical red flags finally leading to the diagnosis. 

Only a patient with self-limited RBFP during adolescence was initially misdiagnosed as recurrent Bell’s palsy; after an ischemic stroke which led the patient to the emergency department, his diagnosis was revised to NS [14]. In another patient, RBFP initially self-resolved with steroid therapy. After two years, NS manifested through other symptoms [17]. Multiple cranial neuropathies were presented in five cases (29%), with a prevalence of acoustic nerve (II (1), III (1), V (2), VI (1), VIII (3) cranial nerves); also clinical, laboratoristic/instrumental signs of meningeal involvement were described in six patients. Leptomeningeal involvement, presented in three patients, often represents a more severe disorder with a risk of hydrocephalus. A patient developed ventricular obstructions by an inflammatory or granulomatous process, making it necessary to perform an emergency ventriculoperitoneal shunt [16]. CSF revealed a protein elevation (>1 g/L) and increased CSF-ACE level, respectively, in 67% and 50% of cases where (CFS data were available on 4/6 and 3/6). Serum ACE levels were normal in more than half of the cases when dosed. Brain imaging was available in nine patients; MRI acquired in the acute phase showed: cranial nerve contrast enhancement (22%); leptomeningeal involvement (33%). Multiple non-enhancing white matter lesions were found in isolated NS patients. No intraparenchymal granulomatous lesions, pituitary gland, or spinal cord involvement were described. Two patients presented with peripheral nervous system (PNS) involvement: EMG demonstrated demyelinating polyneuropathy and a mixed (axonal-demyelinating) pattern [19,20,21,22,23,24,25,26]. No dysautonomic symptoms (orthostatic hypotension, palpitations, hyperhidrosis, gastrointestinal dysmotility, or bowel/bladder dysfunction) or small-fiber neuropathy were described. Tissue biopsies were available for eight patients (47%), significant for sarcoidosis (except in a case in which it was performed one month after steroid therapy) [15]. Transbronchial biopsy was the most frequently performed. No brain biopsies were performed. First-line treatment with corticosteroids was started in all patients and then tapered, except for an isolated NS with demyelinating PNP, treated immediately with intravenous immunoglobulins [19].

Immunosuppressive therapy with methotrexate (MTX) was started only in two patients (12%). Third-line treatment was not described. Follow-up information was available for eight patients: three patients had a complete recovery (remission, R), four patients had a partial improvement without a full recovery (partial recovery, PR), and one patient remained stable (not recovered, NR). No patients died.

Including a literature review by Chappity et al. [8], eight adults with Heerfordt’s syndrome and bilateral facial palsy/plegia have been reported in the literature from January 1970 to March 2022, (Table S1 in Supplementary Materials) summarizes their demographic and clinical characteristics. No prevalence of sex was documented (gender was not reported in four cases). At presentation, the median age was 38 years (ranging from 26 to 52 years old). Four patients (50%) presented with SBF involvement. No details were provided on the chronology of the presentation of bilateral facial deficit in patients reported in the literature review [8]. Lung involvement was prevalent, and it was found in three cases where systemic manifestation data were available. The treatment strategy and follow-up information were available for only half patients: all underwent first-line therapy; an immunosuppressant was started in one patient. Recovery was achieved in all patients (50% of them in R).

## 5. Discussion

Unilateral facial palsy (UFP) is frequent in NS, while BFP only occurs in 15% to 25% of cases [1,4,34]. We confirm that multiple cranial neuropathies are among the most frequent manifestations in NS, presenting in 55% of patients with neurological involvement [34] and more globally in 5–6% of systemic sarcoidosis [9]. Moreover, approximately in half of the suspected NS cases, neurological involvement is the first manifestation that leads to the identification of the disease [32,34]. All of the cranial nerves can be affected, with II, VII, and VIII being the most commonly involved [10], underlying an epineural/perineural granulomatous inflammation or a direct compression by granulomatous inflammation of the leptomeningeal compartment. Our revision confirmed that in 29% of patients, multiple cranial nerve involvement and UFP occur in 20% of the cases among cranial neuropathies in NS, with bilateral involvement reported in 30% of these patients. Our literature revision confirmed that BFP occurs as the first presentation of NS, mostly in young females, and more frequently as a simultaneous presentation (SBFD) than recurrent (RBFP). In 53% of cases, a multiorgan involvement was reported, where BFP was always the first manifestation, representing the first red flag for NS diagnosis.

The prognosis of NS UFP is similar to Bell’s palsy, with complete recovery in about 90% of patients under corticosteroids. However, the resolution of BFP is more often non-simultaneous [10]. Many disorders may cause a BFP, and NS is one of the rarer [2]. Teller and Murphy’s [5] review shows that Lyme disease is responsible for 36% of the cases of BFP. Guillain- Barre syndrome (5%), trauma (4%), sarcoidosis (0.9%), and AIDS (0.9%) are other rarer causes. Diagnosis workup includes serological laboratory tests such as complete blood count, fluorescent treponemal antibody test, HIV test, fasting glucose, erythrocyte sedimentation rate (ESR), Lyme titer, and an antinuclear antibody level measurement. A lumbar puncture could help diagnose an inflammatory process (>50% of the reported patients showed elevated CSF proteins), but a lumbar puncture is also mandatory to rule out other alternative diagnoses. Brain MRI with gadolinium may also reveal cranial nerve enhancement, meningeal involvement, and neoplastic processes, including Internal Acoustic Channel (IAC) and cerebellopontine angle. Frequently, facial diplegia warrants investigations to unveil signs of systemic disorder, such as NS, especially through chest CT and CT-total body PET in selected cases. If clinically indicated, other laboratory investigations may be conducted, such as serum and CSF ACE assay. However, serum and CSF elevated ACE levels are only sometimes found in NS patients, but several studies demonstrated low sensitivity and specificity of this test [34], and it must be stressed that normal ACE results do not exclude the possibility of NS [31,39,40]. Our case showed elevated serum-ACE. However, our literature search confirmed that most patients showed unremarkable serum ACE levels (C SF-ACE was dosed only in six patients with a 50% positivity). NS may manifest in many different ways, making the diagnosis difficult without histologic evidence. Because of the high risks of CNS biopsy, its use is limited to patients with a radiologically confirmed focal lesion in an accessible location or seriously ill patients with rapidly progressive disease. This finding was also confirmed in our literature revision. In patients with less severe disease, it is often more appropriate to look for systemic disease elsewhere in other tissues, as we undertook in the pulmonary district [5,6], even if labial salivary gland [6] and conjunctival [7] could lead to the right diagnosis [11,12].

In the literature, facial diplegia in NS is also described as RBFP, associated with Heerfordt’s syndrome” [8,9,10,11,12]. However, SBFP, defined as the involvement of the opposite side within 30 days of the onset of the first side [18], is only described in 17 adults affected by sarcoidosis without uveoparotitis fever association (Table 1 and Table 2). The clinical presentation of NS can be extremely heterogeneous. The reviewed literature allowed us to recognize that the most frequent clinical scenario of NS’ SBFP is represented by a new onset that develops more frequently in young adult females without a known history of sarcoidosis. Subsequent clinical medical evaluations revealed multiorgan involvement in 81% of cases. If present, SBFP represents one of the first and more frequently recognized manifestations of systemic disease. Neuroimaging, particularly MRI, is usually used to detect and localize neurologic lesions, and it plays a strategical role, especially in the absence of other systemic manifestations of sarcoidosis. Unfortunately, the present case did not show an enhancement to the seventh left cranial nerves, as described previously in NS (Table 1 and Table 2) [14,35,37]. Finally, in our case, acute onset unilateral facial palsy evolving into SBFP, radiologic and serological findings, the response to steroid therapy strongly support the diagnosis of NS as the first manifestation of systemic sarcoidosis [33]; transbronchial lymph node biopsy confirmed the “probable NS” diagnosis by NCCG criteria. In our literature search, only seven patients (41%) fulfilled the criteria for at least probable NS. [32].

As described in the literature, steroids are the most used first-line treatment, then tapered to a minimum maintenance dose. These agents typically suppress inflammation and may relieve acute symptoms. They are recommended in most cases of NS as spontaneous recovery cannot be predicted. An initial dose of prednisone or prednisolone of 20 mg once a day is typically recommended; followed by 5–10 mg once a day to once every other day [38], but in severe cases, high doses of IV methylprednisolone may be used for a few days to achieve a good clinical response in severely ill or deteriorating patients (Table 1 and Table 2) [22,36]. Second-line therapy is generally initiated after steroid tapering and is associated with severe neurological and systemic manifestations [34]. Perhaps reflecting the publication date of some included papers and the lack of long reported follow-up, our search found only two patients treated with methotrexate.

Facial palsy/plegia also represents a neurological manifestation of “Heerfordt’s syndrome” (HS), an acute subtype of sarcoidosis seen in 0.3–1.2% of the cases of sarcoidosis [37]. HS is characterized by facial palsy, parotid gland enlargement, and uveitis associated with low-grade fever. If only two of the three characteristic symptoms are present, an “incomplete Heerfordt’s syndrome” can be defined. HS can be rarely associated with other cranial nerve involvement, particularly affecting the trigeminal nerve [41]. Sarcoid uveitis has a favorable visual outcome since most patients experience mild or no visual impairment [42,43]. However, 2.4 to 10% of patients with sarcoid uveitis develop severe visual impairment [42,43,44,45]. Therefore, ophthalmologic screening is recommended for all patients with newly diagnosed sarcoidosis, even in the absence of symptomatic ocular sarcoidosis. When bilateral VII cranial nerves are involved, ophthalmologic screening and salivatory palpation are mandatory to exclude this specific type of sarcoidosis. 

## 6. Conclusions

Neurosarcoidosis is a great mimicker. A high degree of clinical suspicion and investigations should always be part of the extensive diagnostic workup when multiple cranial nerves are involved. We reported a case of NS that debuted with bilateral facial palsy and reviewed published literature. The biggest limitations of this paper are the small sample size of the historical cases and the lack of complete clinical information and follow-up. More recent NS cohorts often do not clearly specify the unilateral or bilateral facial nerve involvement and therefore make it difficult to draw clear conclusions. However, we believe that even with those limitations, the present review highlights that SBFP/RBFP as the onset of NS is rarely described, representing a diagnostic challenge for clinicians. The prompt recognition of NS and the initiation of appropriate steroid therapy could partially or entirely reverse neurologic sequelae, thus changing the natural progression of the disease. So that when bilateral facial palsy, especially in young adult females, occurs, it is essential to consider the neurological onset of systemic sarcoidosis. Larger studies and revisions are needed to improve knowledge in the field.

## Figures and Tables

**Table 1:** Demographic and clinical properties of patients with bilateral facial palsy/plegia as the onset of NS (Heerfordt’s syndrome cases not included).

N°	Age/Gender/Diagnosis *	Onset/HB (R-L)/Other	Systemic Involvement /Instrumental Findings	Serum //CFS Lab	Serum ACE Onset//FU	Ref
1	48Y/F/Probable NS in P.S I	SBFP/-/ bilat VIII c.n	Lungs/diffused LNP+ -CT: hilar LNP+	N//-	ACE↑//-	[13]
2	25Y/M/Possible NS in P.S II	RBFP recurrences /-/ TIAs, ischemic stroke	Lungs/Kidney/Hematological -CT: LNP+; multiple rounded lungs opacities; BS: nodules	↑Ca^2+^; ↑γ-glob//NA	ACE↑//-	[14]
3	40Y/F/Possible Paradoxical NS TNFα-related in P.S I	SBFD/V-V/ lymphocytic meningitis, anosmia	Lung/Eye/Kidney/Weight loss -Ophthalmological test: papilledema, anterior uveitis -18F-PET-CT: + hilar LNP, parotid	Ca^2+^ ↑; proteinemia↑ /proteinorachia ↑	ACE↑//-	[15]
4	28Y/F/Probable NS in P.S I	Headache, dizziness/ SBFD/-/ bilateral VI c.n/ diffuse leptomeningitis	Lung/Skin/URT -67Ga scintigraphy: hilar LNP+, salivary glands, nasal sinus and cubital fossa	N//proteinorachia ↑ (125 mg/dl); ↑ CSF ACE 2.8 IU F.U.:normal	ACE N//-	[16]
5	33Y/M/Probable NS in P.S II	RBFP recurrences + VI c.n/-/ III c.n/ O.N	Lung/Eye/Skin/Kidney -67Ga scintigraphy: hilar LNP+, salivary glands (uptake +); frontal meningities, lungs,	ESR ↑// NA	ACE↑//-	[17]
6	34Y/F/Probable NS in P:S Grade I	SBFP/V-III/ headache, dysgeusia	Lung/Weight loss -CT: hilar, mediastinal LNP	N// proteinorachia ↑ (125 mg/dl);	ACE N//-	[18]
7	35Y/M/ Probable Isolated NS	SBFP/VI-III/	PSN -EMG: sensorimotor demyelinating PNP -EMG VII nerve: axonal loss, partial denervation	N// lymphocytes ↑; CFS-ACE increased ↑	-//-	[19]
8	24Y/F/NS	SBFP/-/ parotid swelling	Parotid gland	-	-	[20]
9	-/-/NS	SBFP/-/	-	-	-	[21]
10–11	-/-/NS	SBFP/-/	-	-	-	[22]
12	62Y/F/Probable NS in P.S II	SBFP/II-IV/	Lung	N//N	ACE N//-	[23]
13–14	-/-/NS	RBFP/-/	-	-	-	[24] **
15	-/-/NS	SBFD/VI-VI/	-	-	-	[25] **
16	60Y/F/Definite NS in P.S I	SBFD/VI-VI/ aseptic meningitis/ dysarthria, distal limb dysesthesia	Lung/Eye/PNS -Ophthalmological test: bilateral papilledema -67Ga scintigraphy: mediastimal LNP+ (uptake +) -EMG:mixed PNP	N //Aseptic meningitis //CSF ACE-test NA	-//-	[26]
17	43/F/Possible N.S. in P.S Grade I	SBFP/-/-/ Bilateral V c.n Hyposmia, Dysphagia, dysgeusia Hypoesthesia (C8-T12) level (Multiple postganglionic neuropathy—sarcoid polyradiculopathy)	Lung/Eye/PNS -Ophthalmological test: bilateral uveitis -67Ga scintigraphy: mediastimal LNP+ (uptake +) -EMG: F-wave frequency ↓; ↓ bilateral ulnar, median nerve SNAP	N//lymphocytosis, proteinorrachia ↑// CSF ACE-test-	ACE↑//improved	[27]

Abbreviations: ↑: augmented, above the upper limit of normal; ↓ reduced, under the lower limit of normal BS: bronchoscopy; BP: brain biopsy; /-/: unknown; CN: cranial nerve; CT: computed tomography; ESR: erythrocyte sedimentation rate; F: female; F.U.: follow-up; HB Scale: House–Brackmann Scale; LNP+: lymphadenopathy; LNP: lymphonodes; M: male; N: normal; NR: not recovered; NS: neurosarcoidosis; ORL: otorhinolaryngology; PDL: prednisolone; PDN: prednisone; PNP: polyneuropathy; PR: partial recovery; PS: pulmonary sarcoidosis; SBFD: simultaneous bilateral facial di-plegia; R: remission; SBFP/RBFP: simultaneous/recurrent bilateral facial palsy; SCN: subcutaneous nodule;TB: transbronchial; URT: Upper respiratory tract; VP: ventriculoperitoneal; * according to NCCG’ criteria; ** Review article.

**Table 2:** Demographic and clinical properties of patients with bilateral facial palsy/plegia as the onset of NS (Heerfordt’s syndrome cases not included).

N°	Brain Imaging	Biopsy	Treatment Onset//FU	Prognosis/HB (R-L)	Ref
1	Focal leptomenigeal thickening (Gd+)	Soft tissue LNP biopsy +	PDN 60 mg/day //PDN (20 mg/day) + MTX	R/II	[13]
2	Ischemic cerebral stroke	Forearm Kveim test biopsy +	-	R/I-I	[14]
3	Left VII, V, VIII nerves, Gasser's ganglia (Gd+); FU: Normal	TB biopsy—(1M after therapy	Steroid bolus, PDL (1 mg/kg/day) //PDN + MTX (25 mg/week)	NR/-	[15]
4	Left VII, VIII nerves diffuse leptomeningeal, cerebellar cortex (Gd+); severe hydrocephalus -FU: Left VII, VIII nerves (Gd-)	SCN biopsy+	VP shunt, PDL 60 mg/day //PDN 15 mg/day	NA	[16]
5	Temporal leptomeninges (Gd+)	SCN biopsy	PDN// PDN 20 mg/day	NA	[17]
6	N	TB biopsy +	M-PDL(1g/day for 5 days) -> PDN 60 mg //no therapy	R/I-I	[18]
7	Sub/cortical white matter hemispheres lesions	-	IvIgG//-	PR/IV-I * * 3 months after	[19]
8	-	-	-	-	[20]
9	-	-	-	-	[21]
10–11	-	-	-	-	[22]
12	N	TB biopsy+	PDN 30 mg/die * //tapering * low dose due to diabetes	PR/-/* * 1 month after	[23]
13–14	-	-	-	-	[24] **
15	-	-	-	-	[25] **
16	-	Skin and sural nerve biopsy+	-	NA	[26]
17	N	-	M-PDL iv// oral PSL (30 mg/day)	PR/-/	[27]

Abbreviations: ↑: augmented, above the upper limit of normal; ↓ reduced, under the lower limit of normal BS: bronchoscopy; BP: brain biopsy; /-/: unknown; CN: cranial nerve; CT: computed tomography; ESR: erythrocyte sedimentation rate; F: female; F.U.: follow-up; HB Scale: House–Brackmann Scale; LNP+: lymphadenopathy; LNP: lymphonodes; M: male; N: normal; NR: not recovered; NS: neurosarcoidosis; ORL: otorhinolaryngology; PDL: prednisolone; PDN: prednisone; PNP: polyneuropathy; PR: partial recovery; PS: pulmonary sarcoidosis; SBFD: simultaneous bilateral facial di-plegia; R: remission; SBFP/RBFP: simultaneous/recurrent bilateral facial palsy; SCN: subcutaneous nodule;TB: transbronchial; URT: Upper respiratory tract; VP: ventriculoperitoneal; * according to NCCG’ criteria; ** Review article.

## Data Availability

Not applicable.

## Supplementary Materials

The following supporting information can be downloaded at: https://www.mdpi.com/article/10.3390/neurosci3020023/s1. Table S1: Demographic and clinical properties of patients with bilateral facial palsy/diplegia in complete Heerfordt’s syndrome.
