# Supplementary material for: Bilateral Facial Palsy as the Onset of Neurosarcoidosis: A Case Report and a Revision of Literature
Source: NeuroSci. 2022 May 29;3(2):321–31. doi: 10.3390/neurosci3020023 (PMC11523723; doi:10.3390/neurosci3020023)
Supplement: Supplementary file 1 [file neurosci-03-00023-s001.zip › neurosci-1714801-supplementary.pdf]

Supplementary Matherial

Table S1. Demographic and clinical properties of patients with bilateral facial palsy/diplegia in complete Heerfordt's syndrome.

No	Age	Gender	Heerfordt's syndrome manifestations/H.B. Scale	Other Systemic Manifestation	Diagnostic finding	Treatment	Prognosis	References
1	32 Y	Male	SBFP<br>Fever<br>Bilateral parotid swelling<br>Bilateral anterior uveitis	Lung<br><br>LNP+<br>(mediastinal,hilar)	-Serum ACE test ↑<br>-Parotid gland biopsy +	First-line therapy	CR	Srirangaramasamy et al. - 2016 [28]
2	42Y	Female	SBFD/-/<br>Fever<br>Bilateral parotid swelling<br>Unilateral anterior uveitis	Lung/Eye/Skin<br><br>LNP+<br>(mediastinal,hilar)	- Skin biopsy +	First-line therapy	PR	King et al - 2007 [29]
3	26Y	Male	SBFD/-/<br>Fever<br>Bilateral parotid swelling<br>Unilateral anterior uveitis	EMG: proximal demyelination facial nerves.	-	Fist-line therapy<br>Second-line theraphy	CR	Glocker et al. - 1999 [30]
4	52Y	Female	SBFD/IV-IV/<br>Bilateral parotid swelling<br>Unilateral anterior uveitis	Lung<br>LNP+<br>(mediastinal,hilar)	-Serum ACE test ↑<br>- Parotid gland biopsy+	Fist-line therapy	PR (II-I)	Chappity et al - 2015 [8]
5	NA	NA	SBFD/-/<br>Fever<br>Bilateral parotid swelling<br>Anterior uveitis	-	Parotid gland FNA cytology+	-	-	Chappity et al - 2015 [8]
6	NA	NA	SBFD/-/<br>Fever<br>Bilateral parotid swelling<br>Anterior uveitis	-	Parotid gland FNA cytology+	-	-	
7	NA	NA	SBFD/-/<br>Fever<br>Bilateral parotid swelling<br>Anterior uveitis	-	Parotid gland FNA cytology+<br>-Bilateral Gasser's ganglia MRI impairment			
8	NA	NA	SBFD/-/<br>Bilateral parotid swelling<br>Anterior uveitis	-	Parotid gland FNA cytology+	-	-	

CR: complete recovery; /-/: unknown; HB Scale: House Brackmann Scale; FNA: fine-needle aspiration LNP+: lymphadenopathy; PPR: partial recovery; S: skin; SBFD: simultaneous bilateral facial diplegia; SBFP/RBFP: simultaneous/recurrent bilateral facial palsy
